# Preparation of UV-Curable Low Surface Energy Polyurethane Acrylate/Fluorinated Siloxane Resin Hybrid Coating with Enhanced Surface and Abrasion Resistance Properties

**DOI:** 10.3390/ma13061388

**Published:** 2020-03-19

**Authors:** Jianping Zhou, Chunfang Zhu, Hongbo Liang, Zhengyue Wang, Hailong Wang

**Affiliations:** 1 School of Material Science and Engineering, Nanchang Hangkong University, Nanchang 330063, China; zf161162@163.com (J.Z.); Zhuchunfang_123@163.com (C.Z.); 18270890654@163.com (Z.W.); 15732633597@163.com (H.W.); 2 Jianxi Provincial Engineering Research Center for Surface Technology of Aeronautical Materials, Nanchang Hangkong University, Nanchang 330063, China

**Keywords:** fluorosiloxane resin, UV curable, low surface energy, polyurethane acrylate

## Abstract

Low surface energy coatings have gained considerable attention due to their superior surface hydrophobic properties. However, their abrasion resistance and sustainability of surface hydrophobicity are still not very satisfactory and need to be improved. In this work, a series of utraviolet (UV)-curable fluorosiloxane copolymers were synthesized and used as reactive additives to prepare polyurethane acrylate coatings with low surface energy. The effect of the addition of the fluorinated graft copolymers on the mechanical durability and surface hydrophobicity of the UV-cured hybrid films during the friction-annealing treatment cycles was investigated. The results show that introducing fluorosiloxane additives can greatly enhance surface hydrophobicity of the hybrid film. With addition of 2 wt.% fluorosiloxane copolymers, the water contact angle (WCA) value of the hybrid film was almost tripled compared to that of the pristine PU film, increasing from 58° to 144°. The hybrid film also showed enhanced abrasion resistance and could withstand up to about 60 times of friction under a pressure of 20 kPa. The microstructure formed in the annealed film was found to contribute much to achieve better surface hydrophobicity. The polyurethane acrylate/fluorinated siloxane resin hybrid film prepared in this study exhibits excellent potential for applications in the low surface energy field.

## 1. Introduction

Over the past few years, fluoropolymers [1,2,3,4] have attracted much attention due to their many advantages, such as weather resistance, heat resistance, and solvent resistance [5,6]. As the C-F bonds are particularly strong (bond energy of 485 kJ mol^−1^) and the fluorine atoms exhibit low polarizability and strong electronegativity [7], polymers containing a significant fraction of fluorine atoms usually possess excellent chemical and thermal stability [8]. In addition, the fluoroalkyl groups along the molecular chains endow these polymers with unique surface properties, such as adhesion resistance, low friction coefficient, and antifouling properties [9].

Great efforts have been made to develop and optimize fluorinated materials. The relevant research is very extensive, mainly including dielectric performance [10], yellowing resistance, and low volume shrinkage properties of urethane acrylate [11]—the effect of different polymerization methods [12], the influence of perfluoroalkyl chain [13], and the effect of fluorinated siloxane graft copolymer [14]. Especially, some pioneering studies demonstrated that the robust and self-healing ability of the fluorinated materials contribute significantly to the performance of superhydrophobic coatings. For example, Lin et al. [15] prepared superhydrophobic fabrics under 12 KPa and 28,000 cycles of abrasion with wool felt by using a fluoroalkyl silane and crosslinked polydimethylsiloxane (PDMS). Their results showed that the water contact angle of the fabrics remained above 150°, indicating that the superhydrophobic surface character was retained. They also utilized fluorinated-decyl polyhedral oligomeric silsesquioxane (FD-POSS) and hydrolyzed fluorinated alkyl silane (FAS) to prepare superhydrophobic fabrics, which still exhibited superhydrophobic surface even under ten plasma-and-heat cycles [16]. Zhang et al. [17] combined thiol-ene fluorinated siloxane (T-FAS) and hydrophobic fumed silica nanoparticles to prepare superhydrophobic coatings that could withstand approximately 100 abrasion cycles at 45 KPa [18]. It should be noted that, in these examples, the materials were surface-modified materials rather than pure fluoropolymers.

Incorporation of fluorinated groups into acrylate copolymers could bring several desirable properties to the polyacrylate coatings. Depending on their chain length, the contribution of the introduced fluoroalkyl-containing moieties to the material surface properties can be tailored. If the molecular chain of the moieties are too short, they may be buried within the polymer matrix, thus mitigating their contribution [19]. In this aspect, Koji Honda et al. [9] studied the effects of side chain length on the molecular aggregation states. Their results showed that, when the number of fluoromethylene units is less than or equal to six, the fluoroalkyl groups have minimal contribution to the surface energy of the coating. On the other hand, fluorinated polymers containing long fluoroalkyl groups tend to crystallize readily. Therefore, fluorosilicon structures were introduced via graft copolymerization to improve the “self-healing systems” [16,17].

Previous work in our group demonstrated that incorporation of fluorinated siloxane resin into polyurethane could greatly enhance the chemical resistance of the cured coating [20]. In this work, grafted fluorosiloxane resins were employed as reactive additives to prepare UV-curable low surface energy polyurethane acrylate hybrid coatings, and the abrasion resistance and hydrophobicity of the cured coatings during the friction-annealing process were investigated. Three fluorine-containing polymers—namely, poly (hexafluorobutyl acrylate) (P-G01), poly (dodecafluoroheptyl acrylate) (P-G05), and graft copolymer (P-(G01+G05)) with equal molar ratio of G01 and G05—were first synthesized as intermediates, which were further grafted onto glycidyl-modified PDMS (EP-PDMS) to synthesize fluorinated silicone copolymers (G-SP-G01, G-SP-G05, and G-SP-(G01+G05)). The fluoro-containing silicone resins were finally added to commercial polyurethane mixture (DR-U356 + hexafunctional urethane) to prepare the UV-curable low surface energy coatings. To reveal the role of various factors accounting for the abrasion resistance and hydrophobic performance of the film during the friction-annealing cycles, the surface topology and the chemical composition (fluorine content) of the coating were characterized by 3D surface morphology analyzer and X-ray photoelectron spectroscopy (XPS), and the hydrophobicity evolution of the coatings was measured by static water contact angle (WCA). This work provided a strategy to prepare polyurethane acrylate hybrid coating which exhibits great application potential in the area of low surface energy materials.

## 2. Materials and Methods

### 2.1. Materials

Hydrogen-containing polydimethylsiloxane (H-PDMS) was purchased from Shenzhen Chonghuaxin Technology Co., Ltd. (Shenzhen, China). Allyl glycidyl ether (AGE) was supplied by Aladdin Co., Ltd. (Shanghai, China). Isophorone diisocyanate (IPDI) was obtained from Bayer Corporation (Leverkusen, Germany). Acrylic acid (AA), 1-hydroxy-4-methoxyphenol (MEHQ), hydroxyl propyl acrylate (HPA), hexafluorobutyl acrylate (G01), and dodecafluoroheptyl acrylate (G05) were purchased from Haerbin Sunshine Fluorine Silicon Chemical Co., Ltd. (Haerbin, China). Dibutyltin dilaurate (DBTDL), N,N-dimethyl benzyl ammonia (DBA), tetradecyl trimethyl ammonium bromide (MTBA), carbon disulfide, 1-dodecanethiol, chloroform, acetone, and sodium hydroxide were all purchased from Xilong Chemical Co., Ltd. (Shantou, China). Photoinitiator 1173 was obtained from Zhejiang Shindy Dragon Coatings Technology Co., Ltd. (Zhejiang, China). Polyurethane acrylic resin (DR-U356) was obtained from Changxing Materials Co., Ltd. (Suzhou, China) Hexafunctional urethane was purchased from the United States Sartomer Company (Exton, PA, USA). All chemicals were used as received.

### 2.2. Synthesis of RAFT Chain Transfer Agent (RAFT-CTA)

**Scheme 1:**
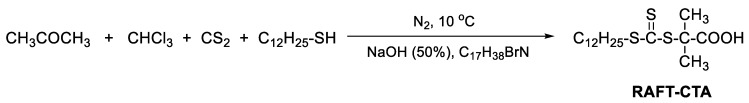
Synthesis of RAFT chain transfer agent.

RAFT-CTA agent was synthesized according to the literature [21] and the synthesis route is shown in Scheme 1. 1-Dodecanethiol (40.38 g, 0.20 mol), acetone (96.2 g, 1.655 mol), and MTBA (2.69 g, 0.008 mol) were first mixed in a jacketed reactor under nitrogen protection and cooled down to 10 °C. Then, 50% sodium hydroxide solution (16.77 g, 0.21 mol) was added over 20 min. The reaction was stirred for an additional 15 min before carbon disulfide (15.21 g, 0.20 mol) dissolved in acetone (20.18 g, 0.345 mol) was added over 20 min, during which time the color of the solution turned red. After 10 min, chloroform (35.625 g, 0.30 mol) was added in one portion, followed by dropwise addition of 50% sodium hydroxide solution (80 g, 1 mol) over 30 min. The reaction was stirred overnight. Afterwards, 300 mL of water was added, followed by 50 mL of concentrated HCl to acidify the aqueous solution. Nitrogen was purged through the reactor with vigorous stirring to evaporate off acetone. The solid was collected with a Buchner funnel and then stirred in 0.5 L of 2-propanol. After filtering off, the 2-propanol solution was concentrated, and the resulting solid was recrystallized from hexanes to afford 45.5 g of yellow crystalline solid.

### 2.3. Synthesis of Glycidyl-Modified PDMS (EP-PDMS)

**Scheme 2:**
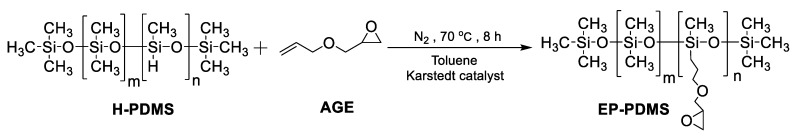
Synthesis of EP-PDMS.

EP-PDMS was synthesized via hydrosilylation reaction between H-PDMS and AGE (Scheme 2). First, AGE (17.1g, 0.15 mol), an equivalent mass of toluene and 300 ppm of Kastedt’s catalyst (with respect to the total mass of reactants) were added into a three-neck round-bottom flask, and the mixture was heated to 65 °C under a nitrogen atmosphere. Then, 50 g H-PDMS was added dropwise to the flask over 30 min while the reaction temperature was increased to 75 °C. The reaction was stirred for 7–8 h and monitored by infrared spectroscopy, in which the disappearance of Si-H peak at 2156 cm^−1^ was considered as the indication of the completion of the reaction. Finally, excess AGE and toluene were removed via vacuum distillation and 50.3 g (a yield of ~75%) of colorless and transparent product was obtained.

### 2.4. Synthesis of Fluorinated Intermediates (PG01, PG05, and P (G01+G05))

**Scheme 3:**
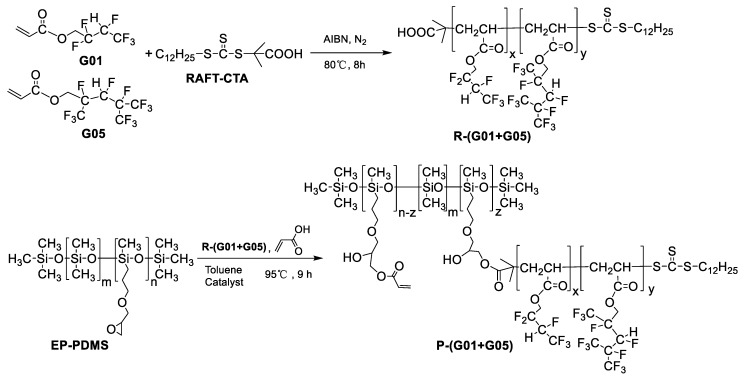
Synthesis of fluorinated intermediary P-(G01+G05).

In this work, three fluorine-containing intermediates (P-G01, P-G05, P-(G01+G05)) were synthesized via grafting RAFT-mediated fluoroacrylate polymers (R-G01, R-G05, R-(G01+G05)) onto EP-PDMS. The synthesis approach adopted for P-SP (G01+G05) is described below (Scheme 3) as an example for the general synthetic strategy to obtain these intermediates.

First, G01 (23.6 g, 0.1 mol), RAFT-CTA (0.364 g, 0.001 mol), and 2,2-azobisisobutyronitrile (AIBN) (0.0405 g, 0.00025 mol) were dissolved in 25 g of toluene in a simple aperture flask equipped with a European-type bubbler. The mixture was subjected to 8 cycles of vacuum evacuation and argon replacement, then heated up to 80 °C and allowed to react for 10 h. Subsequently, G05 (38.6 g, 0.1 mol) was injected into the flask and reacted for an additional 10 h. After being thoroughly purified and dried at 30 °C in vacuum for 72 h, 61.3 g (a yield of ~98%) of a pale-yellow product denoted as R-(G01+G05) was obtained.

Then, 8.16 g EP-PDMS (containing 0.011 mol epoxy group) and 8.16 g toluene were mixed in a three-neck round bottom flask and the mixture was heated up to 65 °C. After 30 min, a mixture of 10 g R-(G01+G05) (0.00367 mol) and 0.454 g DBA was added, and the temperature was increased to 85 °C. The reaction was allowed to proceed for 2 h before 0.259 g AA (0.00734 mol), 0.00186 g MEHQ, 0.0132 g DBA, and 1 g toluene were added into the flask. The reaction mixture was stirred for another 2 h. Finally, the reactants were purified by petroleum ether-acetone method and 11.0 g (a yield of ~60%) of a yellow product denoted as P-(G01+G05) was obtained.

Fluorinated intermediates P-G01 and P-G05 were also synthesized via the same approach as described above.

### 2.5. Synthesis of Blocking Agent (IPDI-HPA)

**Scheme 4:**
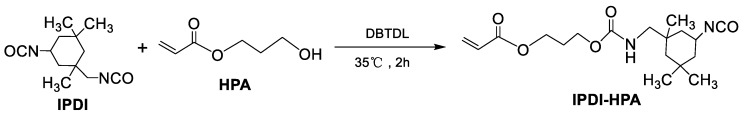
Synthesis of IPDI-HPA.

As shown in Scheme 4, IPDI (11.1 g, 0.05 mol) was first mixed with 0.0003 g of DBTDL in a three-neck flask kept at 0–10 °C. Then, HPA (6.5 g, 0.05 mol) was added dropwise into the flask. The temperature was subsequently raised to 35 °C and the reaction mixture was stirred for an additional 2 h to obtain 16.0 g (a yield of ~91%) of colorless and transparent product denoted as IPDI-HPA.

### 2.6. Synthesis of Fluoropolymers (G-SP-G01, G-SP-G05 and G-SP-(G01+G05))

**Scheme 5:**
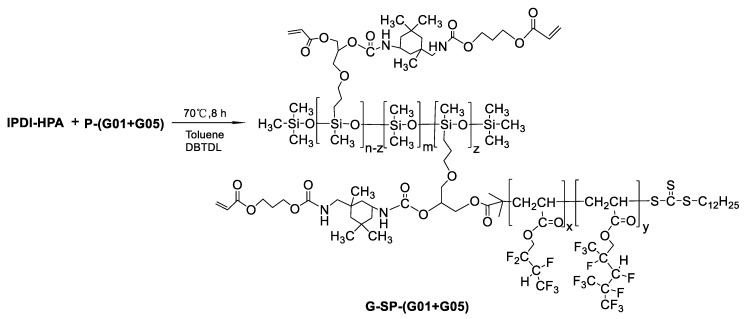
Synthesis of fluoropolymer G-SP-(G01+G05).

First, 15 g P-(G01+G05), 0.5 wt.% DBTDL and MEHQ, 10 g acetone, and 15 g toluene were added into a three-neck flask which was equipped with a thermometer, a condenser, and a constant pressure funnel. The mixture solution was then heated up to 70 °C. Afterwards, the synthesized IPDI-HPA (dissolved in toluene) was added during the reaction. Isocyanate conversion was monitored by Fourier Transform Infrared (FTIR) spectroscopy, in which the disappearance of NCO-associated peak at 2700 cm^−1^ indicated the completion of the reaction. The reactants were purified and dried at 30 °C in vacuum for 72 h and the fluoropolymer product denoted G-SP-(G01+G05) was obtained. Scheme 5 shows the synthesis route. 

Fluorinated polymers G-SP-G01 and G-SP-G05 were synthesized via similar procedures that were adopted for the synthesis of G-SP-(G01+G05) above.

### 2.7. Preparation of UV-Cured Hybrid Coatings of Fluorosiloxane Resin

A series of hybrid coatings was prepared by blending the synthesized fluorosiloxane polymers (G-SP-G01, G-SP-G05, and G-SP-(G01+G05)) with commercial polyurethane mixture (DR-U356 + hexafunctional urethane) and curing under UV irradiation. The preparation of the hybrid coating with G-SP-(G01+G05) addition is described below as a representative procedure that was used for the preparation of all hybrid coatings of fluorosiloxane resins in this work.

In a typical procedure, 0.2 g G-SP-(G01+G05) copolymer was first mixed with 7 g DR-U356 and 2.8 g hexafunctional urethane. Afterwards, 2 g acetone and 0.4 g 1173 (photo-initiator) were added. The mixture was coated on a tinplate (dimension: 100 mm × 50 mm × 0.2 mm) which was prepolished by 240-mesh and 600-mesh sandpaper using an 80-µm-wide wire rod applicator. The wet film was stored at 60 °C for 30 min and then cured by UV light. All samples were cured using UV lamp (UVAH400a; Moisten mechanical and Electrical Co, Ltd., Dongguang, China). The average thickness of the cured film was measured to be 6–8 µm.

### 2.8. Friction-Annealing Experiment

To assess the mechanical durability and sustainability of the surface hydrophobicity of the UV-cured hybrid coatings, a friction-annealing experiment was adopted in our studies. During the experiment, the sample film was first abraded 20 times with 800-mesh sandpaper under a pressure of 20 KPa (called a friction treatment process), and then put into an oven to undergo an annealing treatment at 120 °C for 30 min (called an annealing treatment process). The two successive treatment processes form a friction-annealing cycle. In each cycle, XPS and WCA measurements were conducted immediately after the film underwent a friction treatment process or an annealing treatment process.

### 2.9. Characterization

FTIR measurements of the synthesized fluorinated polymeric products were performed using a VERTEX70 spectrometer (Bruker, Germany) in transmission mode. To prepare the sample for spectrum recording, diluted solution of polymer samples was dropped onto the potassium bromide (KBr) discs and kept at ambient temperature for a few minutes to evaporate the solvent. For each sample, 32 scans were acquired and averaged, with a scanning range of 400–4000 cm^−1^ and a resolution of 4 cm^−1^. All spectra were manipulated by using 9-point smoothing, baseline correction, and normalization.

Proton nuclear magnetic resonance (^1^H-NMR) spectra were recorded on a UAVANCEIII 400 MHz spectrometer (Santa Clara, CA, USA). All spectra were collected at room temperature with a 0.5% *(w/v)* sample solution in CDCl_3_ or DMSO-d_6_. During the spectra collecting, zg45 pulse sequence was used and 24 scans were performed for each sample.

Contact angle measurements were conducted with a Drop Shape Analyzer (DSA20, Krüss, Germany). The measurement was conducted at room temperature (20 °C) with 0.6-μL water droplets. For each sample film, five independent measurements were performed, and the results were averaged.

The roughness of the hybrid coating surface was monitored using an UP-24 3D universal optical profilometer (Rtec Instruments, Sanjose, CA, USA). CF mode was adopted for the observation with a 100× lens and 15-fps confocal frame rate. Averaged roughness was measured by Gwyddion analysis software (version 2.55) [22] based on the collected images with an optical resolution of 0.16 μm.

X-ray photoelectron spectroscopy (XPS) measurements were carried out to investigate the fluorine content at the film surface by using an Escalab 250 Xi spectrometer (Thermo Scientific, Waltham, MA, USA) equipped with a monochromatic Al-Kα radiation (1486.74 eV) and a PHOIBOS 150 hemispherical electron analyzer (SPECS, Berlin, Germany). The scans were performed with an x-ray power of 25 W, a take-off angle of 45°, and a pass energy of 20 eV. The base pressure at the analysis chamber was below 2 × 10^−7^ pa. 10 scans were accumulated for each sample. CasaXPS software (version 2.3.16, Casa Software Ltd, Wilmslow, UK) was used to process and analyze the XPS data. The binding energy charge correction was performed by setting the adventitious carbon at 284.7 eV.

## 3. Results and Discussion

### 3.1. Characterization of the Synthesized EP-PDMS and Fluorinated Siloxane Copolymers

**Figure 1:**
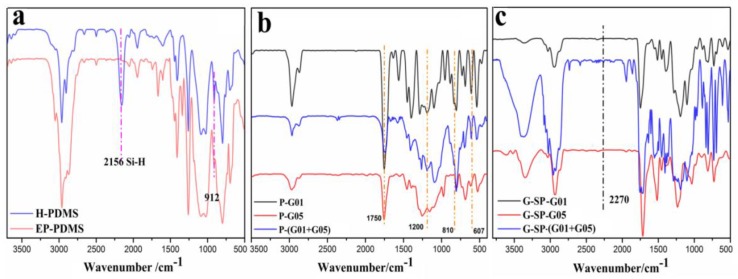
FTIR spectra of (**a**) H-PDMS and EP-PDMS; (**b**) P-G01, P-G05, and P-(G01+G05); (**c**) G-SP-G01, G-SP-G05, and G-SP-(G01+G05).

The chemical structures of the functionalized intermediates (EP-PDMS; P-G01, P-G05, and P-(G01+G05); G-SP-G01, G-SP-G05, and G-SP-(G01+G05)) were first confirmed by FTIR. Figure 1a shows the spectra of H-PDMS and EP-PDMS. The strong peak at 2156 cm^−1^ assigned to Si-H [23] disappeared in the EP-PDMS curve, indicating the completion of hydrosilylation. In addition, a new weak absorption peak appeared at around 912 cm^-1^ which is attributed to the vibration of the epoxy ring introduced by the AGE monomer [24,25]. Figure 1b shows the spectra of P-G01, P-G05, and P-(G01+G05). Strong peaks at 1700 cm^−1^, 1200 cm^−1^, 810 cm^−1^, and 607 cm^−1^ assigned to C=O, –CF_3_, C=C, and –CF_2_, respectively [20,25], were observed in the P-G01, P-G05, and P-(G01+G05) traces, indicating that the fluorine-containing intermediates and AA were successfully grafted onto the molecular chains of EP-PDMS. Figure 1c shows the spectra of G-SP-G01, G-SP-G05, and G-SP-(G01+G05). The peaks at 2270 cm^−1^ assigned to –NCO [20,24] were undetectable in all traces, indicating the completion of coupling of IPDI-HPA to the polymers P-G01, P-G05, and P-(G01+G05).

**Figure 2:**
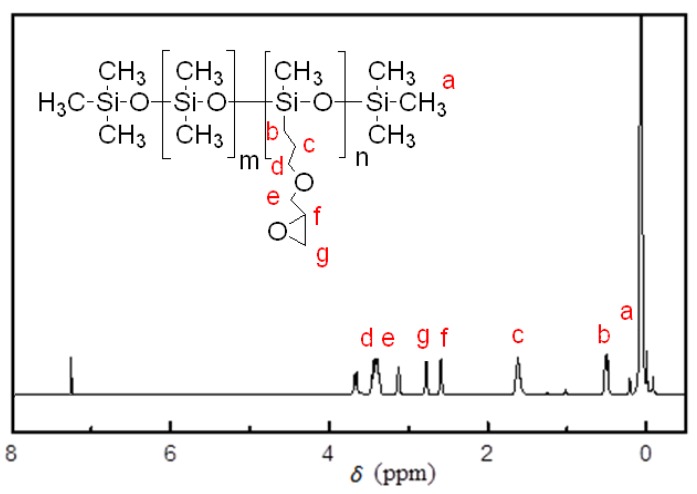
^1^H-NMR spectra of the synthesized EP-PDMS.

Figure 2 shows the H-NMR spectrum of EP-PDMS in CDCl_3_ solvent. The strong signals that appeared around *δ* 0 ppm belong to the silicon methyl protons [24]. The specific signal at *δ* 0.49 ppm (*b*) comes from the methylene protons formed after hydrosilylation reaction, and the signals appeared at *δ* 2.58 ppm (*f*); *δ* 2.77 ppm (*g*) belongs to the protons of the epoxy groups [24]. The appearance of these peaks confirmed the successful synthesis of EP-PDMS.

**Figure 3:**
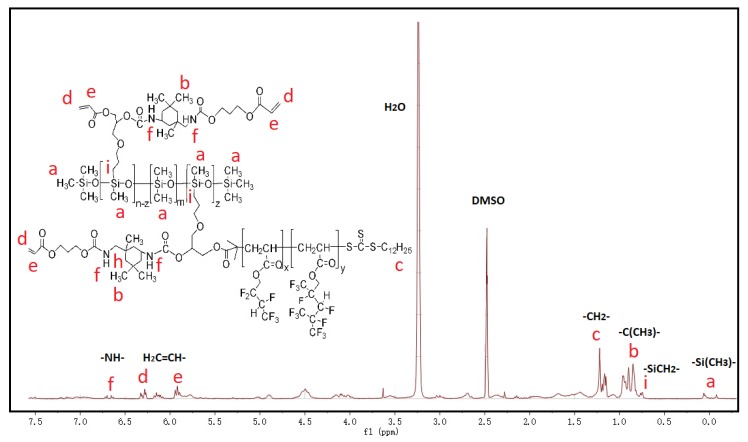
^1^H-NMR spectra of the fluorinated siloxane copolymer G-SP-(G01+G05).

Figure 3 shows the ^1^H-NMR spectrum of the synthesized fluorinated siloxane copolymer G-SP-(G01+G05) in DMSO-d6 solvent. The characteristic resonance signals of the double bonds bearing on the grafted side chains appear at *δ* 5.92 ppm (*e*), 6.28 ppm (d) [23,24]; the signal at *δ* 6.7 ppm (*f*) corresponds to the -NH- protons [20,24] formed via the coupling reaction between IPDI-HPA and the fluorinated intermediates. The proton signal at *δ* 0.75 ppm (i) belongs to the methylene [24] that linked to the PDMS backbone via hydrosilylation. The signals at around *δ* 0 ppm belong to the silicon methyl proton on the backbone [24]. Due to the shielding effect of the side chains, the signal strength of methyl group is greatly weakened. In addition, the signals of methyl protons that linked to the IPDI rings locate at around *δ* 0.90 ppm (*b*) [23,25]. The occurrence of these characteristic signals indicates that the desired fluorinated siloxane copolymer was successfully synthesized.

### 3.2. Mechanical Durability of the UV-Cured Hybrid Coatings with Different Fluorinated Polymers Addition

**Figure 4:**
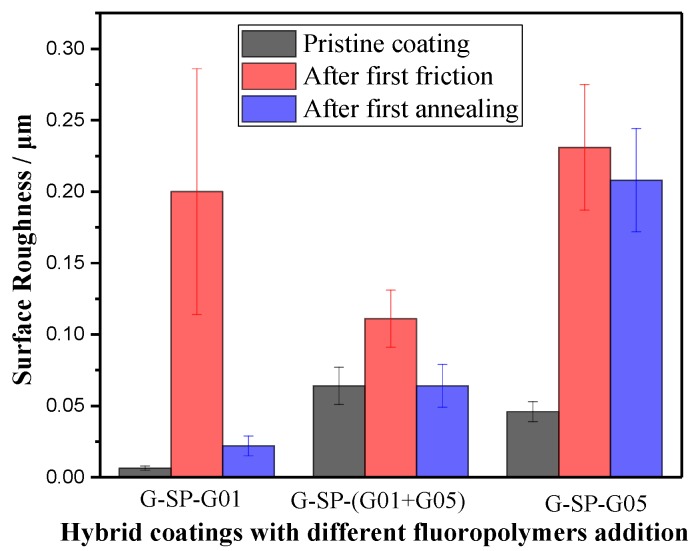
The surface roughness of the hybrid coatings during the first friction-annealing cycle.

The low surface energy coatings in use often need to be scrubbed when soiled. Therefore, the mechanical durability of these coatings is very important for practical applications. Since the surface of the coating is often roughened by repeated scrubbing, the change of the surface roughness after friction treatment can be regarded as an indication of the mechanical stability of the hybrid coatings. Shown in Figure S1 in supplementary is the surface morphology of the hybrid coatings with different fluorosiloxane copolymers addition recorded by the UP-24 3D universal optical profilometer, and the statistical values of the surface roughness for each film during the first friction-annealing cycle are shown in Figure 4.

As can be seen, the initial height value of the two hybrid films with G-SP-G01 or G-SP-G05 addition was 0.006 μm and 0.046 μm, respectively. After the first friction treatment, the height values of these two films increased to 0.200 μm and 0.231 μm, respectively. However, the topological features on the film with G-SP-(G01+G05) addition did not vary markedly, only increasing from 0.064 μm to 0.111 μm. The results show that, after the first friction treatment process, the hybrid film with 2 wt.% G-SP-(G01+G05) addition exhibited the lowest roughness. Moreover, the surface roughness of the film has the smallest change. Therefore, it can be inferred that the mechanical stability of the film is better than the other two kinds of hybrid films.

After the subsequent annealing treatment, it is apparent that the three kinds of hybrid films all exhibited a decrease in the surface roughness as expected. This is because the thermal treatment could help restore the smooth surface due to the relaxation, diffusion, and migration of molecular chain segments under heating.

### 3.3. Surface Composition of the UV-Cured Hybrid Coatings with Different Fluorinated Polymers Addition

**Figure 5:**
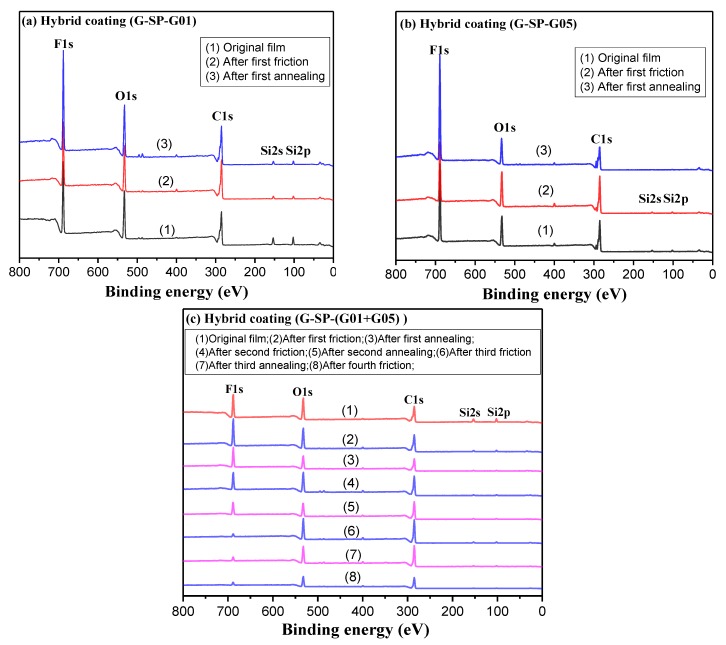
XPS traces of the three hybrid coatings during the friction-annealing treatment process: (**a**) hybrid coating with 2 wt.% G-SP-G01 addition; (**b**) hybrid coating with 2 wt.% G-SP-G05 addition; (**c**) hybrid coating with 2 wt.% G-SP-(G01+G05) addition.

Chemical composition is another important factor that influences the hydrophobicity of the films. The results of XPS measurements of the UV-cured hybrid coatings with different fluorinated silicone polymers G-SP-G01, G-SP-G05, and G-SP-(G01+G05) addition are shown in Figure 5. The signals that occurred at 285.1, 533.1, and 688.1 eV were attributed to C1s, O1s, and F1s, respectively; and the signals at 153.1 eV and 101.1 eV were attributed to Si2s and Si2p.

**Figure 6:**
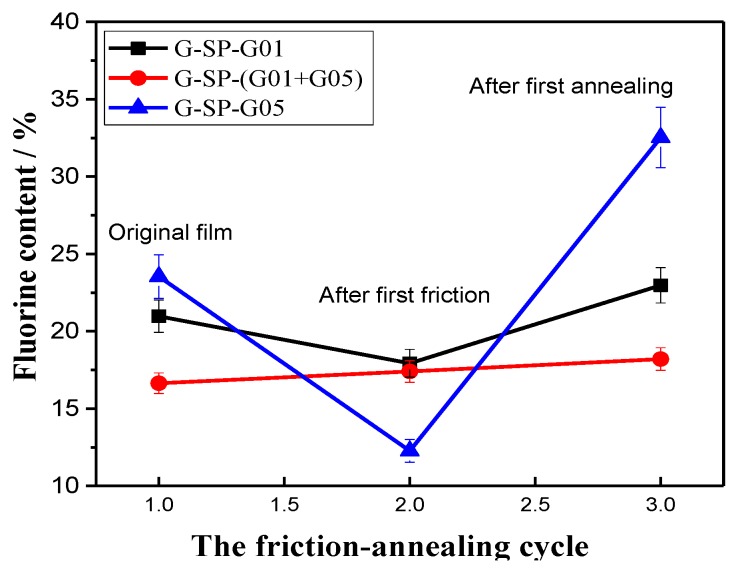
The change in fluorine content on the surface of the hybrid coatings during the first friction-annealing cycle.

Figure 6 demonstrates the change of the fluorine content at the surface of the three hybrid films during the friction-annealing cycle. Despite the measurement error, a remarkable trend was clearly exhibited. As can be seen, the initial fluorine content at the surface of the hybrid films with G-SP-G01, G-SP-G05, and G-SP-(G01+G05) addition was 20.97%, 23.54%, and 17.4%, respectively. After the first friction treatment process, the fluorine content at the surface of the hybrid film with the addition of G-SP-G01 and G-SP-G05 dropped significantly, decreasing to 17.93% and 12.29%, respectively; while that of the hybrid film with G-SP-(G01+G05)-addition just showed a very slight increase. After the subsequent annealing treatment, the hybrid film with G-SP-G01 addition exhibited a moderate increase in the fluorine content (increasing from 17.93% to 22.98%), but the fluorine content of the film with G-SP-G05 addition showed a dramatic increase (increasing from 12.27% to 32.53%). The decrease of the fluorine content of the two hybrid films after friction treatment could be attributed to the destroying of the crosslinked structure and removal of the chemical composition from the film surface because of abrasion. It also reflected the existence of the concentration gradient of fluorine element along the surface into the internal layer of the film. Actually, the introduced fluorinated additives have weak miscibility with the PU matrix network. Therefore, microphase separation will occur during the UV curing process, and the fluoroalkyl segments tend to migrate to the outer layer of the hybrid film, resulting in an enrichment of fluorine element at the film surface. When a further thermal treatment was applied to the hybrid films, the motion of the chain segments of the introduced fluoroalkyl moieties was liberated and the migration to the film surface was greatly promoted, leading to a notable increase of fluorine content at the film surface and the reconstruction of the hydrophobicity of the surface [26]. By comparison, the hybrid film with G-SP-G05 addition exhibited a larger increase in the fluorine content as compared to that of the film with G-SP-G01 addition. This is presumably because the length of molecular side chain of the fluorinated polymer G-SP-G05 is larger than that of G-SP-G01, enabling the side-chain segments of the former easier to move and migrate to the outer surface.

Strikingly, the fluorine content of the hybrid coating film with G-SP-(G01+G05) addition kept very stable during the whole friction-annealing treatment cycle, only exhibiting a very gentle increase of 0.8% (from 17.4 to 18.2%). The results demonstrate that this hybrid film has stable chemical composition at the surface during the friction-annealing treatment process, which is important for the application of low surface energy coatings.

### 3.4. Surface Hydrophobicity of the UV-Cured Coatings

Surface hydrophobicity is the key parameter to be considered for the application of low surface energy coatings. The contact angle of the cured coatings during the first friction-annealing cycle was measured and the data were shown in Table 1.

**Figure 7:**
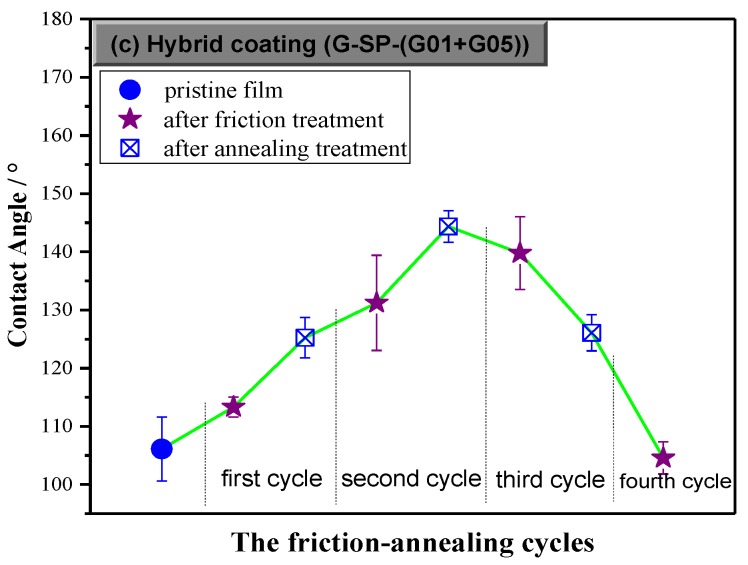
Hydrophobicity of the hybrid coating with 2 wt.% G-SP-(G01+G05) addition in four friction-annealing cycles.

As can be clearly seen, the WCA values of the pristine hybrid films were all larger than that of the neat PU film, indicating that introducing fluoropolymers greatly increased the hydrophobicity of the matrix resin. After the first friction treatment, the WCA values of the coatings all increased. Since the fluorine content of hybrid coatings decreased (as shown in Figure 7) and the surface roughness of the hybrid coatings all increased after friction (as shown in Figure 5), it could be inferred that the increase of the surface roughness plays a major role in the enhancement of the surface hydrophobic property at this stage.

The data in Table 1 also show that the WCA values of the coatings increased when the coatings underwent a further thermal treatment. As the fluorine content of the coatings increased while the surface roughness decreased in this process, the surface hydrophobicity was mainly affected by the chemical composition at the surface. The hybrid film with G-SP-G05 addition exhibited a mostly hydrophobic surface after the annealing treatment, which could be ascribed to its highest fluorine content at the surface.

### 3.5. Sustainability of the Surface Hydrophobicity of the Hybrid Film with G-SP-(G01+G05) Addition

Sustainability of the hydrophobicity is required and crucial for the long-term service life of low surface energy coatings. Based on the above results, the hybrid film with G-SP-(G01+G05) exhibited superior mechanical stability and satisfactory surface hydrophobic performance during the first friction-annealing cycle. Therefore, this film was selected to further run several friction-annealing cycles to explore its capability in retaining the surface hydrophobicity.

As shown in Figure 7, the WCA values of the hybrid film kept increasing and reached the peak value of 144° after two friction-annealing treatment cycles. From the beginning of the third cycle, the WCA value of the film started to decline, but it was still larger than that of the pristine film even after the third annealing treatment process, indicating the film could endure three friction treatments (equaling to 60 times of scrubbing with 800-mesh sandpaper under 20 KPa pressure).

**Figure 8:**
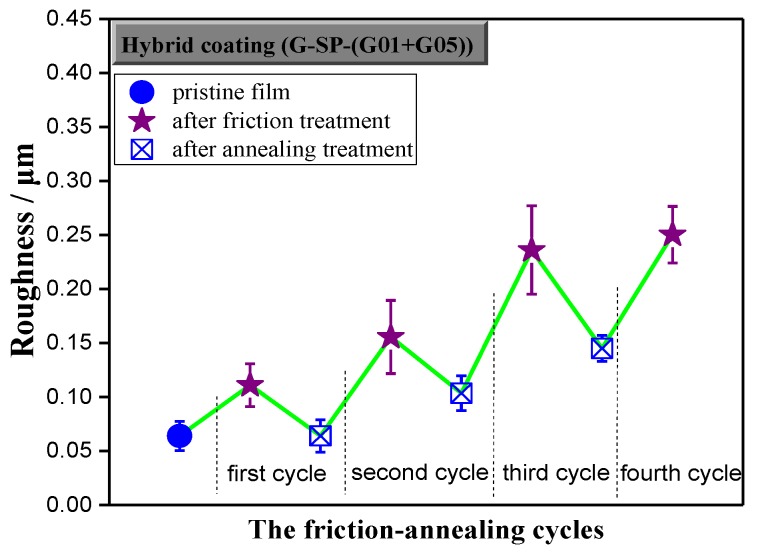
Roughness change for hybrid coating film with 2 wt.% G-SP-(G01+G05) during the abrasion–annealing cycle.

**Figure 9:**
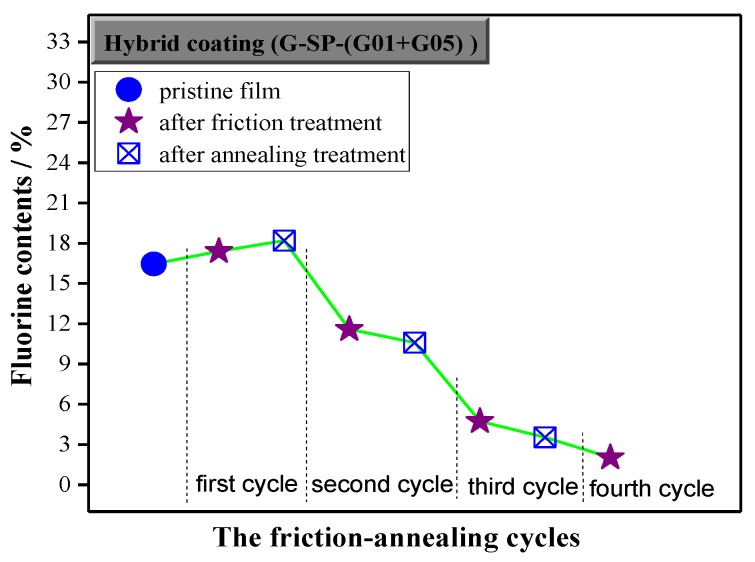
Change in fluorine fraction content at the surface for film with 2 wt.% G-SP-(G01+G05) during the abrasion–annealing cycle.

The surface roughness and chemical composition are two main factors that influence the film surface hydrophobicity. To clearly elucidate the role of these factors in affecting the hydrophobic performance of the hybrid film, the surface morphology and chemical compositions of the film during the multiple friction-annealing treatment cycles were investigated by RTEC and XPS. The results in Figure 8 demonstrate that the surface roughness of the hybrid film increased continuously in each friction treatment process or annealing treatment process. When compared with the last friction treatment, the roughness of the annealed film decreased slightly as expected. The data in Figure 9 reveal that the fluorine content at the surface of the hybrid film kept decreasing after each friction treatment or after each annealing treatment. However, it is worth noting that the surface fluorine content of the annealed film kept almost unchanged when compared to that after the last friction treatment, which is different from the general rule that the fluorine content of annealed film would be higher than that of the last friction-treated film [27]. The reason for this phenomenon is not clear at the moment, but it has been speculated to be closely related to the mechanical properties (such as compactness) of the hybrid film. The UV-cured coatings usually possess high crosslinking density and hardness when compared to traditional coatings that are prepared from conventional thermal curing technique [28], especially when multifunctional monomers are involved, which may hinder the mobility of the fluoroalkyl chains.

It is well known that roughness enhances the intrinsic hydrophobicity of a coating. Herein, the surface roughness of the hybrid film took on a zigzag growth (Figure 8) and the fluorine content kept decreasing (Figure 9) during the friction-annealing treatment processes, however, the WCA profile did not exhibit the same zigzag growth pattern, indicating the annealing treatment contributed much to the WCA value of the annealed film. Since the roughness of the annealed film decreased and the fluorine content kept almost the same as compared to that of the last friction-treated film, the increase of WCA value of the annealed film should be resulted from the effect of annealing on the surface microstructure of the film. Usually, more refined surface microstructure could be obtained after the annealing treatment process, which is favorable for the surface hydrophobicity. The surface hydrophobicity of the intrinsic fluoropolymer coatings had been stabilized by successive thermal annealing [29] or even significantly improved when a suitable annealing treatment was employed [30]. With addition of a little amount of fluorinated diblock copolymer, hydrophobic properties of the coatings could also be increased through further thermal treatment, which was assumed to be attributed to the orientation of the fluoro-containing ligands and the transition of surface morphology [31]. Moreover, annealing could improve the antifouling properties of the coatings prepared from fluoropolymer brushes and even yield better long-term antifouling behavior under harsh environments [32]. However, it should be pointed out that, in most cases, the reregulation of surface composition (enrichment of fluorine element) and evolution of surface morphology (regular micro-/nano-structure) occurs simultaneously during the thermal treatment process.

## 4. Conclusions

Novel UV-curable fluorinated siloxane resins were successfully synthesized and further used as reactive additives to prepare hybrid coatings with low surface energy. The results show that introducing fluorosiloxane copolymers into polyurethane acrylate coating could greatly enhance the surface hydrophobicity of the hybrid film. With 2 wt.% fluorinated copolymer G-SP-(G01+G05) addition, the UV-cured hybrid coating was found to exhibit excellent mechanical durability and superior hydrophobicity. It could undertake about 60 times of scrubbing with 800-mesh sandpaper under a pressure of 20 KPa, and the maximum WCA value could reach up to 144° during the friction-annealing treatment process. Except for roughness and fluorine content, surface microstructure was found to contribute much to achieve better surface hydrophobicity. In summary, this study elucidated the possibility to prepare polyurethane acrylate coatings with enhanced performance in mechanical stability and surface hydrophobicity via hybridization approach. The as-prepared coatings have good potential for applications in the low surface energy industry.

## Figures and Tables

**Table 1:** Contact angle for the UV-cured coatings during the first friction-annealing cycle.

	Neat PU Film	Hybrid Film with G-SP-G01 Addition	Hybrid Film with G-SP-G05 Addition	Hybrid Film with G-SP-(G01+G05) Addition
Pristine film	58° ± 2.8°	122° ± 2.5°	115° ± 4.5°	105° ± 4.2°
After first friction treatment	77° ± 2.3°	120° ± 2.7°	122° ± 3.5°	114° ± 2.5°
After first annealing treatment	82° ± 3.5°	119° ± 2.0°	146° ± 3.1°	125° ± 4.5°

## Supplementary Materials

The following are available online at https://www.mdpi.com/1996-1944/13/6/1388/s1, Figure S1: Surface morphology imaging showing the pristine coating, following the first friction treatment and following subsequent first annealing treatment for hybrid coating samples: (**a**) hybrid coating with 2 wt.% G-SP-G01 addition; (**b**) hybrid coating with 2 wt.% G-SP-(G01+G05) addition; (**c**) hybrid coating with 2 wt.% G-SP-G05 addition.
