# Supplementary material for: Preparation of UV-Curable Low Surface Energy Polyurethane Acrylate/Fluorinated Siloxane Resin Hybrid Coating with Enhanced Surface and Abrasion Resistance Properties
Source: Materials (Basel). 2020 Mar 19;13(6):1388. doi: 10.3390/ma13061388 (PMC7143720; doi:10.3390/ma13061388)
Supplement: Supplementary file 1 [file materials-13-01388-s001.pdf]

materials

Supplementary Materials

Preparation of UV-Curable Low Surface Energy Polyurethane Acrylate/Fluorinated Siloxane Resin Hybrid Coating with Enhanced Surface and Abrasion Resistance Properties

Jianping Zhou, Chunfang Zhu, Hongbo Liang *, Zhengyue Wang, and Hailong Wang

School of Material Science and Engineering, Nanchang Hangkong University, Nanchang 330063, China; zf161162@163.com (J.Z.); Zhuchunfang_123@163.com (C.Z.); 18270890654@163.com (Z.W.); 15732633597@163.com (H.W.)
* Correspondence: lhongbo@nchu.edu.cn

Received: 12 November 2019; Accepted: 17 March 2020; Published: date

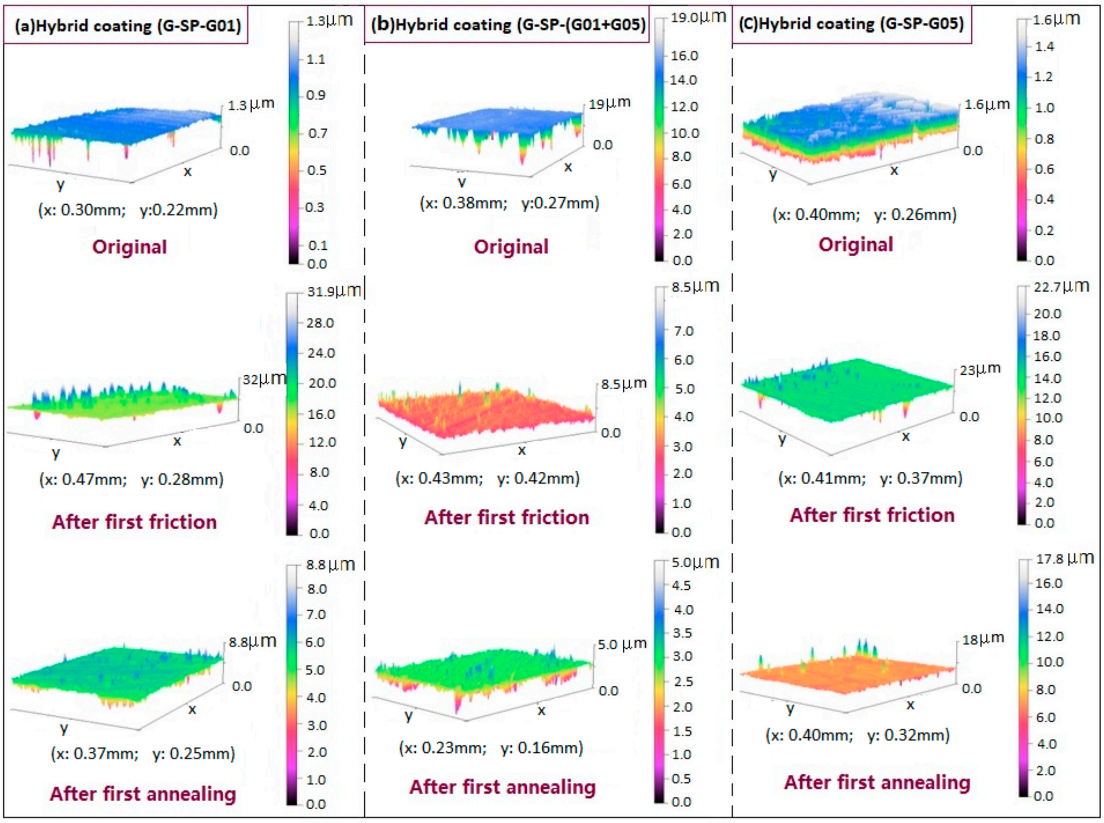

Figure S1. Surface morphology imaging showing the pristine coating, following the first friction treatment and following subsequent first annealing treatment for hybrid coating samples: (**a**) hybrid coating with 2 wt.% G-SP-G01 addition; (**b**) hybrid coating with 2 wt.% G-SP-(G01+G05) addition; (c) hybrid coating with 2 wt.% G-SP-G05 addition

© 2020 by the authors. Submitted for possible open access publication under the terms and conditions of the Creative Commons Attribution (CC BY) license (http://creativecommons.org/licenses/by/4.0/).
